# Protein Kinase A Is Essential for Invasion of Plasmodium falciparum into Human Erythrocytes

**DOI:** 10.1128/mBio.01972-19

**Published:** 2019-10-08

**Authors:** Mary-Louise Wilde, Tony Triglia, Danushka Marapana, Jennifer K. Thompson, Alexei A. Kouzmitchev, Hayley E. Bullen, Paul R. Gilson, Alan F. Cowman, Christopher J. Tonkin

**Affiliations:** a The Walter and Eliza Hall Institute of Medical Research, Parkville, Melbourne, Victoria, Australia; b Department of Medical Biology, The University of Melbourne, Melbourne, Victoria, Australia; c Utrecht University, Utrecht, Netherlands; d Burnet Institute, Melbourne, Australia; e Department of Microbiology, Monash University, Melbourne, Australia; University of Geneva

**Keywords:** AMP-activated kinases, *Plasmodium falciparum*, host cell invasion, malaria

## Abstract

Understanding the mechanisms behind host cell invasion by Plasmodium falciparum remains a major hurdle to developing antimalarial therapeutics that target the asexual cycle and the symptomatic stage of malaria. Host cell entry is enabled by a multitude of precisely timed and tightly regulated receptor-ligand interactions. Cyclic nucleotide signaling has been implicated in regulating parasite invasion, and an important downstream effector of the cAMP-signaling pathway is protein kinase A (PKA), a cAMP-dependent protein kinase. There is increasing evidence that P. falciparum PKA (PfPKA) is responsible for phosphorylation of the cytoplasmic domain of P. falciparum apical membrane antigen 1 (PfAMA1) at Ser610, a cAMP-dependent event that is crucial for successful parasite invasion. In the present study, CRISPR-Cas9 and conditional gene deletion (dimerizable cre) technologies were implemented to generate a P. falciparum parasite line in which expression of the catalytic subunit of PfPKA (PfPKAc) is under conditional control, demonstrating highly efficient dimerizable Cre recombinase (DiCre)-mediated gene excision and complete knockdown of protein expression. Parasites lacking PfPKAc show severely reduced growth after one intraerythrocytic growth cycle and are deficient in host cell invasion, as highlighted by live-imaging experiments. Furthermore, PfPKAc-deficient parasites are unable to phosphorylate PfAMA1 at Ser610. This work not only identifies an essential role for PfPKAc in the P. falciparum asexual life cycle but also confirms that PfPKAc is the kinase responsible for phosphorylating PfAMA1 Ser610.

## Abstract (precis)

Malaria continues to present a major global health burden, particularly in low-resource countries. Plasmodium falciparum, the parasite responsible for the most severe form of malaria, causes disease through rapid and repeated rounds of invasion and replication within red blood cells. Invasion into red blood cells is essential for P. falciparum survival, and the molecular events mediating this process have gained much attention as potential therapeutic targets. With no effective vaccine available, and with the emergence of resistance to antimalarials, there is an urgent need for the development of new therapeutics. Our research has used genetic techniques to provide evidence of an essential protein kinase involved in P. falciparum invasion. Our work adds to the current understanding of parasite signaling processes required for invasion, highlighting PKA as a potential drug target to inhibit invasion for the treatment of malaria.

## INTRODUCTION

Malaria is a devastating disease in resource-poor countries, continuing to have one of the most severe global burdens of all infectious diseases. Malaria is caused by *Plasmodium* parasites, the most lethal being Plasmodium falciparum (1). Once transmitted to humans via the bite of an infected female *Anopheles* mosquito, P. falciparum sporozoites migrate to the liver, where they differentiate and divide into thousands of liver merozoites that are eventually released into the bloodstream. In the blood, merozoites invade and replicate inside erythrocytes before release back into the bloodstream. The occurrence of repeated rounds of this process of asexual amplification leads to the destruction of red blood cells (RBCs) and is responsible for the clinical symptoms of malaria (2).

Erythrocyte invasion by P. falciparum is a tightly regulated process involving a series of receptor-ligand interactions. Following initial contact with erythrocytes, high-affinity interactions occur between micronemal proteins on the merozoite surface and a range of different receptors (3). The merozoite then reorients such that the apical end contacts the erythrocyte membrane, where apically located rhoptry organelles are triggered to secrete their contents. These include a complex of proteins stored in the rhoptry neck, termed RONs, which are injected into the erythrocyte membrane and serve as receptors for merozoite entry (4, 5). P. falciparum apical membrane antigen 1 (PfAMA1) on the merozoite surface engages the RON complex, and this interaction forms the basis of the so-called “moving junction” (MJ) between the parasite and erythrocyte membranes (4, 5). The current model suggests that the MJ is then dragged to the basal end of the merozoite via the activity of the actomyosin motor, thereby pushing the merozoite into the erythrocyte (3).

In order to correctly trigger this molecular cascade of invasion into erythrocytes, P. falciparum must be able to sense and respond to the surrounding environment. However, the mechanisms that mediate environmental sensing and the signal transduction events that drive the processes of invasion have been poorly understood to date. Several signaling pathways have been implicated in triggering P. falciparum invasion, including Ca^2+^ and cyclic nucleotide signaling (6–9). Ca^2+^ signaling has been the best-characterized signaling pathway in *Plasmodium* spp. and has been shown to be crucial at several points throughout the invasion process, including parasite egress and invasion (7, 8, 10). Furthermore, studies in the related parasite Toxoplasma gondii have enabled further dissection of Ca^2+^ signaling at several points in the parasite life cycle (9, 11–13). Following egress, Ca^2+^ signaling is crucial for the release of adhesins from microneme organelles (6, 10). The cAMP and cGMP second messengers have also been implicated as important signaling molecules in various stages of the P. falciparum life cycle (10, 14–17). A rise in the cytosolic cGMP level has been shown to trigger downstream Ca^2+^ signaling, which in turn stimulates release of micronemal proteins involved in egress (10). cAMP signaling, on the other hand, has been largely implicated in parasite invasion and is thought to mediate intracellular Ca^2+^ mobilization prior to invasion (14).

The principal downstream effector of cAMP-signaling is protein kinase A (PKA), a 40.2 kDa protein composed of a single catalytic subunit of P. falciparum PKA (PfPKAc) and a single regulatory subunit containing two cAMP-binding sites (PfPKAr) (18–20). PfPKAc has been implicated in merozoite invasion, with increasing evidence indicating that this kinase is responsible for phosphorylating P. falciparum apical merozoite antigen 1 (PfAMA1), a key protein on the merozoite surface that is required for invasion (21–23). However, PfAMA1 is differentially phosphorylated and dephosphorylated at various residues in its cytoplasmic domain and it is unclear how these signals influence merozoite invasion (23). Recent studies in T. gondii revealed that T. gondii PKAc1 (TgPKAc1) is responsible for dampening Ca^2+^ signaling following host cell invasion and that genetic depletion of TgPKAc1 renders parasites unable to regulate their intracellular Ca^2+^ levels, leaving them more sensitive to cGMP-induced Ca^2+^ signaling (24). Another study described a premature egress phenotype following genetic depletion of TgPKAc1 and correlated this with a disruption in the cross talk between cAMP and cGMP signaling (25). What remains clear is that a complex but poorly understood form of interplay exists between the cyclic nucleotide and Ca^2+^ signaling pathways.

Despite the knowledge that cAMP-dependent phosphorylation is important for successful parasite invasion, the precise role of this signaling cascade during P. falciparum invasion is unclear. Here we examine the role of PfPKAc, the major downstream effector of cAMP-dependent phosphorylation in P. falciparum infection of red blood cells. Through CRISPR/Cas9-mediated gene disruption of the catalytic subunit, we show that PfPKA is essential for parasite survival and confirm that it is the kinase responsible for phosphorylation of PfAMA1 at Ser610. Through live-imaging studies, we demonstrate a crucial role for PfPKAc in merozoite invasion of erythrocytes, a finding in line with a recent publication (26). This work highlights the essentiality of cAMP-dependent protein phosphorylation in enabling entry of parasites into the host cell.

## RESULTS

### PfPKAc is essential for parasite survival.

**FIG 1:**
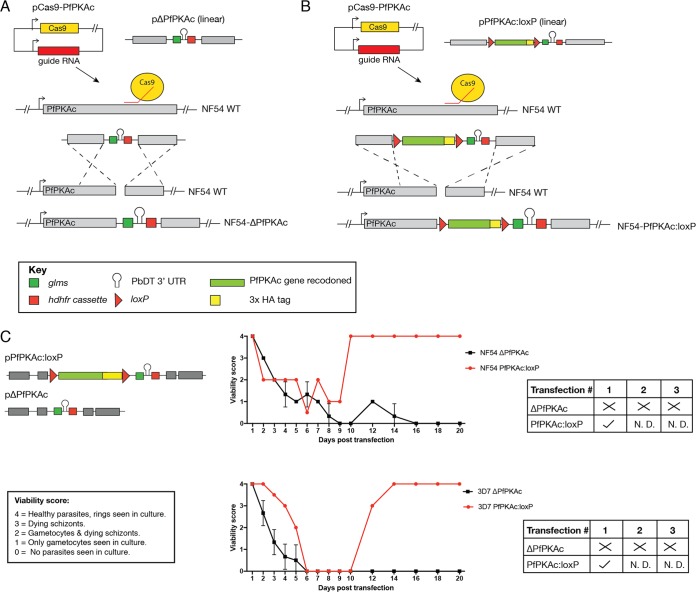
PfPKAc is essential for parasite viability. (A) Schematic of PfPKAc gene disruption (ΔPfPKAc). (B) Schematic of conditional knockout line generation (PfPKAc:loxP). The same backbone was used for generation of both constructs used for transfection. (C) Following transfection, parasites were monitored and given viability scores each day. Tables summarize outcomes of each transfection. ×, no parasite survival; ✓, parasite survival; N.D., not done. In both NF54 and 3D7, gene disruption was unsuccessful as no parasites were observed after 30 days in culture (ΔPfPKAc, *n* = 3). PfPKAc:loxP transfection was successful in both 3D7 and NF54 and was not repeated.

To determine the importance of PfPKAc, we attempted to disrupt the *pfpkac* gene. This was performed by cotransfecting the PfPKAc guide plasmid with pΔPKAc (Fig. 1A). In tandem, an allelic swap methodology was employed to reconstitute the *pfpkac* gene with a 3× hemagglutinin (HA) epitope tag and place it under dual conditional regulation via the insertion of two *loxP* sites, as well as a *glmS* riboswitch (Fig. 1B). Following transfection with the two constructs generated, parasite cultures were monitored daily and assigned a viability score based on healthy parasite morphology and the presence of gametocytes, often seen during the drug selection process. Parasites from both lines (3D7 and NF54) were unable to expand on WR99210 (WR) selection after transfection with the knockout construct ΔPfPKAc, while WR-resistant PfPKAc:loxP-transfected parasites were observed 9 to 10 days after transfection (Fig. 1C). To confirm that PfPKAc gene deletion was not achievable, transfection and selection for stable knockout were performed an additional two times in both 3D7 and NF54 without success, despite the high efficiency of integration afforded by the use of CRISPR/Cas9. Together, these data suggested that PfPKAc is essential for maintenance of P. falciparum intraerythrocytic growth.

### DiCre-mediated excision of the PfPKAc gene.

**FIG 2:**
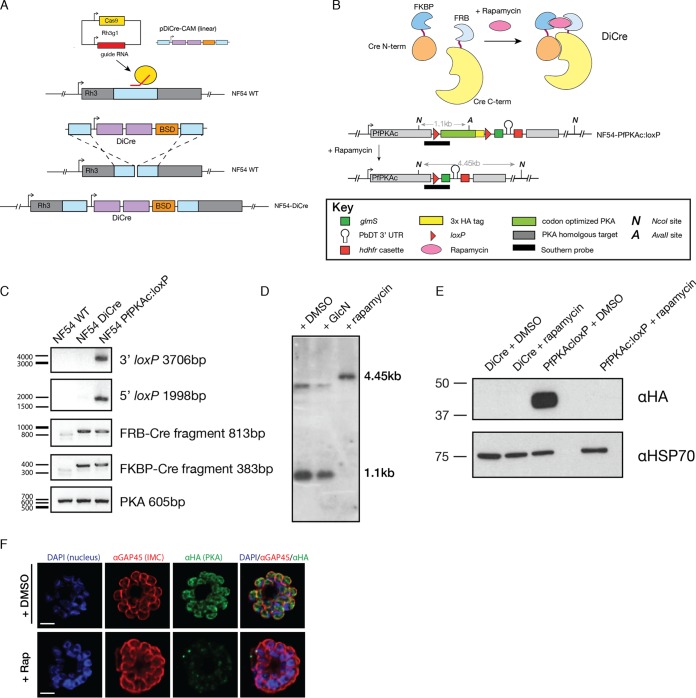
Conditional regulation of PfPKAc. (A) Parental parasite lines expressed DiCre, which was inserted into the *rh3* locus using Cas9-stimulated repair with pDiCre-CAM, a plasmid encoding the two halves of Cre recombinase as well as a blasticidin resistance cassette. Conditional knockout PfPKAc:loxP parasites were generated in the DiCre-expressing line. (B) Schematic of the dimerizable Cre recombinase (DiCre) system. The N-terminal and C-terminal fragments of Cre recombinase were conjugated to one of two rapamycin binding proteins, the FK506-binding protein (FKBP12) or the FKBP12-rapamycin binding (FRB) domain of FKBP12-rapamycin-associated protein (FRAP). Addition of rapamycin enables heterodimerization of the two inactive Cre components via interactions of rapamycin-binding proteins to restore Cre activity. Cre recombinase recognizes the two 34-bp *loxP* sites inserted into the *pkac* locus and excises the gene sequence between them, rendering *pkac* inactive. Restriction sites for Southern blotting (NcoI and AvaII) are shown as well as the resulting sizes of the expected RNA fragments expressed in kilobase pairs (kb). (C) Integration of DiCre and of both *loxP* sites was confirmed by PCR. A fragment (605 bp) at the 5′ end of the *pka* gene was amplified as a loading control. (D) Southern blot analysis of NcoI/AvaII digests confirmed excision of PfPKAc following the addition of rapamycin. NF54 PfPKAc:loxP parasites were cultured for 48 h in the presence of DMSO, GlcN, or rapamycin prior to genomic DNA harvest. (E) Western blot showing PfPKAc-HA expression levels following rapamycin treatment. NF54 DiCre or PfPKAc:loxP parasites were cultured for 72 h in the presence of DMSO or GlcN. Lysates were prepared from late-stage schizonts. Anti-HA was used to detect PfPKAc expression, and anti-PfHSP70 was used as a loading control. (F) Widefield imaging of NF54 PfPKAc:loxP schizonts labeled with anti-GAP45, anti-HA, and DAPI. PfPKAc-HA shows partial peripheral and cytoplasmic localization. Scale bars represent 2.5 μm.

To understand the function of PfPKAc, we used dimerizable Cre recombinase (DiCre) and ribozyme technology (27, 28). Parental lines were generated to stably express integrated DiCre from the *rh3* pseudogene locus (Fig. 2A) (29). DiCre is inactive in its resting state (Fig. 2B), where the two fragments of the Cre enzyme are tagged with either FK506-binding protein (FKBP12) or FKBP12-rapamycin-binding (FRB) domain of FKBP12-rapamycin-associated protein (FRAP). Rapamycin treatment induces interactions between FKBP12 and FRB and therefore reconstitutes the activity of Cre. The active Cre recombinase can then catalyze the recombination of genetic sequences between *loxP* sites (27). The presence of DiCre, as well as both *loxP* sites, in genomic parasite DNA was confirmed by PCR (Fig. 2C). Genomic integration and protein HA tagging were also confirmed in the 3D7 parasite line (Fig. S1 in the supplemental material). Activation of DiCre by rapamycin treatment led to the recognition of *loxP* sites flanking the region of the *pfpkac* gene containing the active site and to excision of this fragment (Fig. 2B). We then monitored rapamycin-dependent gene excision by Southern blotting to deduce the efficiency of the excision event (Fig. 2D). Rapamycin treatment performed within one intraerythrocytic cycle from the ring stage through the schizont stage of development resulted in very efficient *pfpkac* excision with no observation of the unexcised gene. As expected, no excision was observed following glucosamine (GlcN) treatment, which activates the *glmS* ribozyme to mediate self-cleavage of mRNA posttranscriptionally (Fig. S2A and B in the supplemental material). To understand if genetic deletion could be achieved early enough to prevent protein expression, we monitored PfPKAc-HA protein levels by anti-HA immunoblotting. The PfPKAc-HA fusion protein could not be detected after one intraerythrocytic growth cycle in the presence of rapamycin, demonstrating complete knockdown (Fig. 2E). Immunofluorescence (IF) analysis of control PfPKAc-HA parasites showed a cytoplasmic and slightly peripheral membrane localization of the PfPKAc-HA fusion protein around the developing merozoites compared to PfGAP45 (Fig. 2F). Addition of rapamycin led to a specific loss of PfPKAc-HA signal without affecting the localization of PfGAP45 (Fig. 2F, bottom panels).

10.1128/mBio.01972-19.1 FIG S1 Confirmation of transfected 3D7 parasite line. (A) Integration of DiCre and of both *loxP* sites was confirmed by PCR. A fragment (605 bp) at the 5′ end of the *pka* gene was amplified as a loading control. (B) HA tagging of PfPKAc was confirmed by Western blotting, with a CS2 parasite line in which plasmepsin V (PMV) is triply HA tagged used as a positive control. HSP70 was used as a loading control. Download FIG S1, TIF file, 2.1 MB. Copyright © 2019 Wilde et al. 2019 Wilde et al. This content is distributed under the terms of the Creative Commons Attribution 4.0 International license.

10.1128/mBio.01972-19.2 FIG S2 Ribozyme-mediated knockdown of PfPKA. (A) Schematic of the *glmS* ribozyme in PKAloxP parasites. The ribozyme is inserted after the coding region so that it is maintained in the expressed mRNA. In PKAloxP, it is inserted after the second *loxP* site. When added, GlcN binds to the ribozyme, resulting in self-cleavage of the mRNA. The mRNA is then degraded, leading to knocking down of protein expression. (B) Immunoblot showing PfPKAc-HA levels in NF54 PfPKAc:loxP parasites following addition of GlcNc. Parasites were cultured for 72 h in the presence of GlcN at 0.5, 1, 2.5, or 5 mM. Anti-HSP70 antibodies were used as a loading control. (C) Immunoblot showing PfPKAc-HA levels in DiCre and PfPKAc:loxP parasites following addition of DMSO, rapamycin, or GlcN or of rapamycin and GlcN in combination. Densitometry was performed using ImageJ software, and values were calculated relative to HSP70 controls. (D) Light microscopy images of Giemsa-stained NF54 PfPKAc:loxP parasites following treatment with DMSO, rapamycin, or GlcN or with rapamycin and GlcN for 48 h (cycle 1) or 96 h (cycle 2). Download FIG S2, TIF file, 2.7 MB. Copyright © 2019 Wilde et al. 2019 Wilde et al. This content is distributed under the terms of the Creative Commons Attribution 4.0 International license.

### PfPKAc is required for parasite growth.

**FIG 3:**
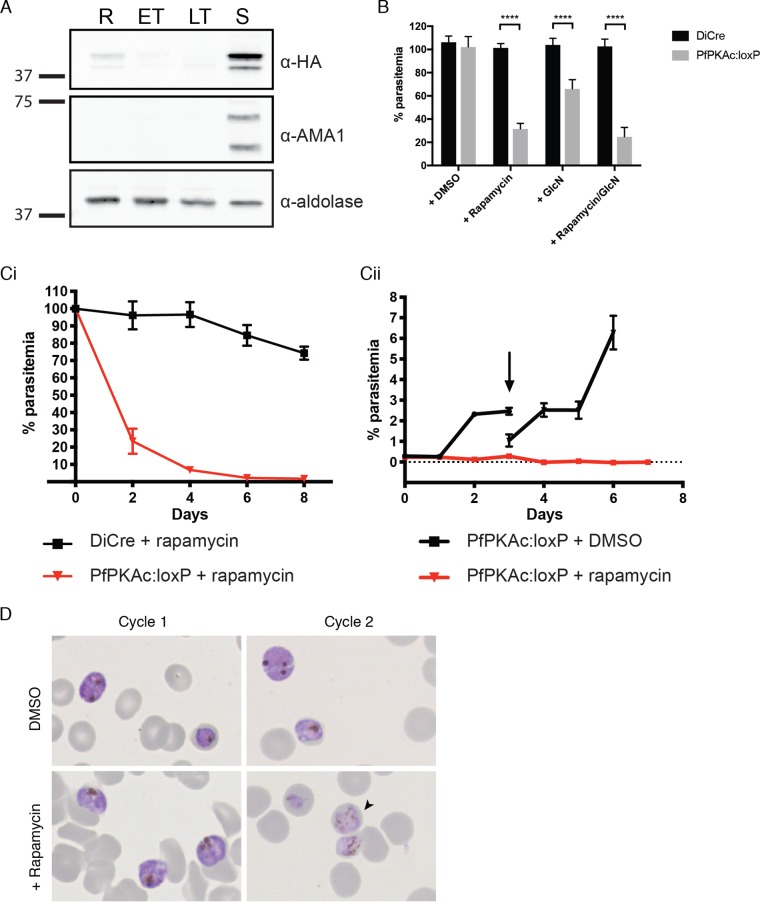
PfPKAc is required for P. falciparum growth. (A) Immunoblot showing Pf*PKA*-HA expression in NF54 PfPKAc:loxP rings (R), early trophozoites (ET), late trophozoites (LT), and schizonts (S). Anti-AMA1 was used as a late-stage-specific control, while anti-aldolase was used as a loading control. (B) Growth of parasites in the presence of rapamycin and GlcN. NF54 WT or PfPKAc:loxP parasites were cultured for 72 h under standard conditions or with the addition of DMSO, rapamycin, or GlcN or of both rapamycin and GlcN. Trophozoites were harvested and stained with ethidium bromide, and parasitemia was determined by FACS analysis. Growth is expressed relative to NF54 PfPKAc:loxP parasites grown under standard conditions. Data are expressed as means ± standard deviations (SD). ****, *P* < 0.0005 (Sidak’s multiple-comparison test). (C) (Panel i) Growth of NF54 PfPKAc:loxP parasites over 4 cycles compared to wild-type parasites in the presence of rapamycin. At the end of each intraerythrocytic growth cycle, late schizonts were harvested for FACS analysis to determine parasitemia levels. Growth is expressed relative to DMSO controls. (Panel ii) Parasites were added to fresh erythrocytes at 0.2% parasitemia, and growth was monitored over 4 cycles. The arrow indicates where the parasites were split under the DMSO treatment conditions. Data are presented as means ± SD (*n* = 3). (D) Light microscopy of Giemsa-stained NF54 PfPKAc:loxP parasites shows that rapamycin-treated parasites developed normally to schizonts within the first cycle but failed to progress into the next intraerythrocytic cycle. The parasites that remained after a second cycle of growth appeared to be dying or undergoing gametocytogenesis (black arrowhead).

We next tested whether PfPKAc is essential for the complete intraerythrocytic cycle. Immunoblotting across the parasite intraerythrocytic life cycle for the PfPKAc-HA fusion protein revealed expression limited to the schizont stage, which was similar to the expression profile of invasion ligand PfAMA1 (Fig. 3A). To understand the role of PfPKAc during intraerythrocytic growth, parental (DiCre) and PfPKAc:loxP parasites were cultured for one intraerythrocytic cycle from ring-stage to schizont-stage parasites in the presence of rapamycin, GlcN, or dimethyl sulfoxide (DMSO), and growth was monitored by flow cytometry. Significant reductions in parasitemia were observed in PfPKAc:loxP parasites following treatment with rapamycin or GlcN or rapamycin and GlcN in combination, while no effect was observed in rapamycin-treated parental parasites. Rapamycin treatment resulted in an almost 70% reduction in parasitemia of PfPKAc:loxP parasites, while GlcN treatment led to a reduction in parasitemia of approximately 30%, indicating that the growth phenotype due to DiCre-mediated gene excision represents greater efficiency than that resulting from activation of the *glmS* ribozyme (Fig. 3B). Furthermore, rapamycin treatment resulted in a greater level of knockdown at the protein level than was seen with GlcN (Fig. S2C in the supplemental material).

Due to the greater knockdown efficiency resulting from DiCre activation, we employed this method to characterize the function of PfPKAc. To assess the impact of loss of PfPKAc function over an extended period of culture, PfPKAc:loxP parasites were grown in the presence of rapamycin for 8 days (constituting 4 intraerythrocytic cycles). Parasitemia was assessed by flow cytometry and expressed as a percentage relative to the results seen with DMSO-treated controls. While parental DiCre parasites showed a slight reduction in growth after 4 cycles of rapamycin treatment, the growth rate of PfPKAc:loxP parasites decreased dramatically after 1 cycle of rapamycin treatment and continued to decline, reaching >98% after 9 days (Fig. 3, panel i). Upon rapamycin-induced excision, the PfPKAc:loxP parasites failed to expand over 4 intraerythrocytic growth cycles, while DMSO-treated PfPKAc:loxP parasites were able to expand normally at each cycle (Fig. 3, panel ii). These results are consistent with PfPKAc being essential for parasite growth and viability. During this period, parasites were monitored by light microscopy of Giemsa-stained parasites. Parasites visualized in the first cycle of rapamycin treatment showed healthy schizonts; however, after one round of egress and reinvasion in the second cycle, very few parasites could be seen, which is consistent with the rapid decrease in parasitemia observed by flow cytometry, and those present appeared to be dying or undergoing gametocytogenesis (Fig. 3D). This observation was mirrored following GlcN treatment (Fig. S2D in the supplemental material). Collectively, these data suggest a role for PfPKAc acting at the late stages of the intraerythrocytic parasite life cycle, during either merozoite egress or invasion.

### PfPKAc is required for merozoite invasion of erythrocytes but not egress.

**FIG 4:**
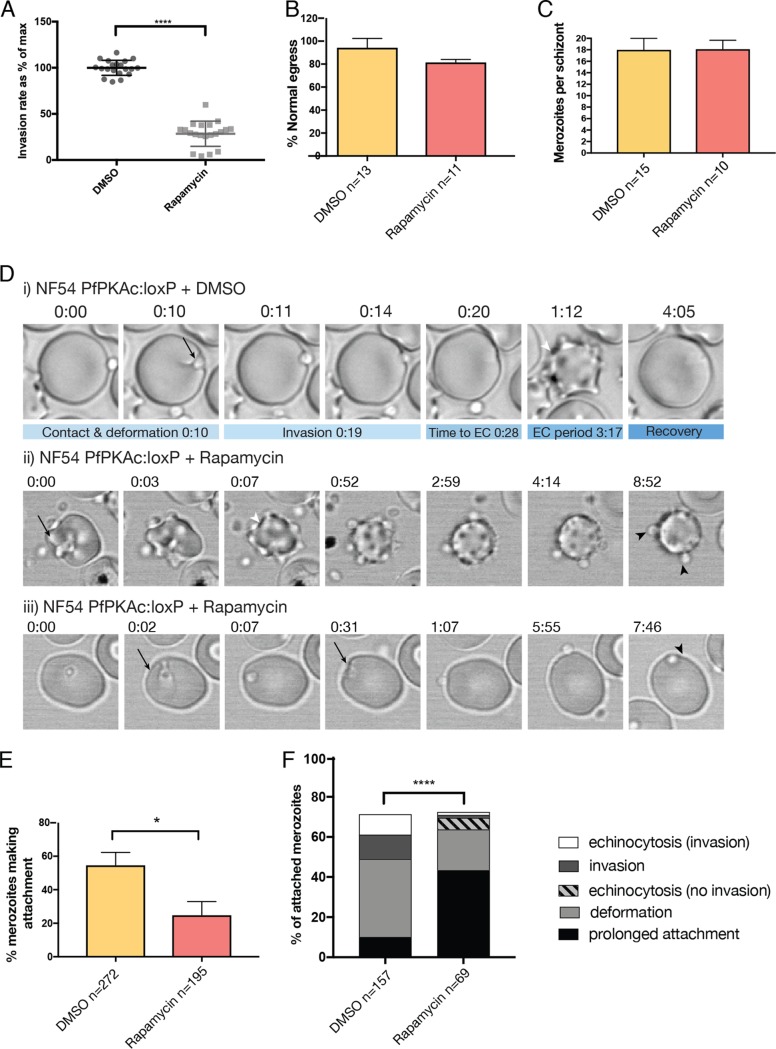
PfPKAc is required for successful merozoite invasion. (A) NF54 PfPKAc:loxP parasites were treated with rapamycin for one growth cycle, and merozoites were purified and added to fresh red blood cells in an invasion assay. Data represent results determined for pooled technical replicates from 4 independent experiments. ****, *P* < 0.0005 (unpaired *t* test). (B) Parasites undergoing egress were imaged by live microscopy. “Normal egress” was defined as complete release of motile merozoites in less than 3 s, with no clumping of merozoites. (C) Live imaging revealed no defect in schizont development. Schizont morphology was determined by counting merozoites per schizont undergoing egress in live-imaging experiments. (D) (Panel i) Selected still images from Video S1 showing a PfPKAc:loxP DMSO-treated merozoite successfully deforming the erythrocyte membrane (black arrow), invading, and triggering echinocytosis (white arrowhead). (Panel ii) Stills from Video S2. (Panel iii) Stills from Video S3. Rapamycin-treated merozoites can still deform the erythrocyte membrane and stimulate echinocytosis (black arrow and white arrowhead, respectively); however, they remain attached for prolonged periods without invading (black arrowheads). (E) PfPKAc-deficient parasites attached to erythrocytes at a lower frequency than controls. Data are presented as means ± SEM, *=*P* < 0.05 (unpaired *t* test). (F) Outcomes were tracked for all merozoites that made initial attachment, showing prolonged attachment durations and reduced invasion frequencies by PfPKAc-deficient parasites. *, *P* < 0.05; ****, *P* < 0.00005 (chi-square test). Quantification of live-imaging experiments was performed on pooled data from 14 independent experiments for each set of conditions, collected over three different days.

To understand at what stage during egress and invasion PfPKAc functions, we performed live-cell imaging (Fig. 4). Parasite egress is extremely rapid, taking as little as 400 ms to completely release merozoites into the environment (30). We defined “normal” egress as occurring in less than 3 s, resulting in a wide dispersal of highly motile merozoites without clumping (see Video S1 in the supplemental material). Quantification of results of live-imaging experiments showed that there was no defect in egress following PfPKAc knockdown (fig. 4B). Depletion of PfPKAc did not affect the number of merozoites released from each schizont (Fig. 4C).

Since previous work had implicated PfPKAc in merozoite invasion (14, 21, 23, 26), we determined whether the growth defect observed following PfPKAc knockdown was due to its role in this stage. PfPKAc:loxP merozoites were purified following treatment with DMSO or rapamycin, and invasion assays were performed to determine their capacity to invade erythrocytes (31). Due to the very short half-life of merozoites following purification, it is difficult to accurately determine merozoite concentrations before adding them to this assay, so this must be done retrospectively. This resulted in a large spread of rates of invasion in the mutant which probably reflected variability in the number of merozoites per erythrocyte as well as their fitness in each particular experiment. Invasion rates were calculated as percentages of RBCs invaded × [(RBCs per microliter)/(merozoites per microliter)] (31). On average, the PfPKAc-deficient line showed a 71.5% reduction in the proportion of merozoites invading following rapamycin treatment (Fig. 4A).

Given the defect reflected in the merozoite invasion rate, we next wanted to identify the moment at which PfPKAc-deficient parasites arrest their entry into host erythrocytes. Typical merozoite invasion occurs as a progression of distinct morphological events beginning with deformation of the erythrocyte membrane shortly after the merozoite makes contact (32, 33). Merozoite deformation probably acts to reorient the apical tip of the merozoite onto the erythrocyte surface. Erythrocyte penetration then proceeds, and, once it is fully internalized, a period of erythrocyte echinocytosis follows where the host cell develops surface protrusions. After several minutes, the erythrocyte recovers its normal biconcave shape and the merozoite differentiates into an amoeboid ring-stage parasite (33). Each of these invasion stages involves specific molecular events (32–34). To determine how PfPKAc functions during invasion, we used live imaging to follow the fate of each merozoite following schizont rupture. This revealed a reduction in the percentage of merozoites forming an attachment with new erythrocytes, as defined by contact lasting longer than 500 ms (Fig. 4E; *P* = 0.0158). Merozoites that attached to erythrocytes were still able to trigger membrane deformation (Fig. 4D, panels ii and iii, black arrows; see also Video S2 and Video S3) and even echinocytosis irrespective of rapamycin treatment (Fig. 4D, panel ii, white arrowhead; see also Video S2). However, only 1.44% of PfPKAc-deficient merozoites were able to invade compared with 12.1% of DMSO-treated merozoites, consistent with results from invasion assay experiments (Fig. 4F). We note that of the PfPKAc-deficient merozoites that attached to erythrocytes, 43.5% maintained prolonged attachment attachment to the erythrocyte surface (Fig. 4D, black arrowheads), in contrast with 10.2% of DMSO-treated parasites (Fig. 4F). A total of 5.7% of PfPKAc-deficient merozoites were also observed stimulating prolonged (greater than 120 s) echinocytosis even though they did not invade, a phenomenon not seen in DMSO-treated PfPKAc:loxP parasites (Fig. 4D and F). PfPKAc therefore plays an essential role in enabling the merozoite to attach and penetrate the erythrocyte following apical reorientation.

10.1128/mBio.01972-19.4 VIDEO S1 Egress and reinvasion of erythrocytes by DMSO-treated NF54 PfPKAc:loxP parasites. Download Movie S1, AVI file, 0.2 MB. Copyright © 2019 Wilde et al. 2019 Wilde et al. This content is distributed under the terms of the Creative Commons Attribution 4.0 International license.

10.1128/mBio.01972-19.5 VIDEO S2 Rapamycin-treated PfPKAc:loxP parasites stimulate echinocytosis but cannot invade. Download Movie S2, AVI file, 2.8 MB. Copyright © 2019 Wilde et al. 2019 Wilde et al. This content is distributed under the terms of the Creative Commons Attribution 4.0 International license.

10.1128/mBio.01972-19.6 VIDEO S3 Rapamycin-treated PfPKAc:loxP parasites deform the erythrocyte membrane but cannot invade. Download Movie S3, AVI file, 3.8 MB. Copyright © 2019 Wilde et al. 2019 Wilde et al. This content is distributed under the terms of the Creative Commons Attribution 4.0 International license.

### PfPKAc knockdown leads to a loss of PfAMA1 phosphorylation at S610.

**FIG 5:**
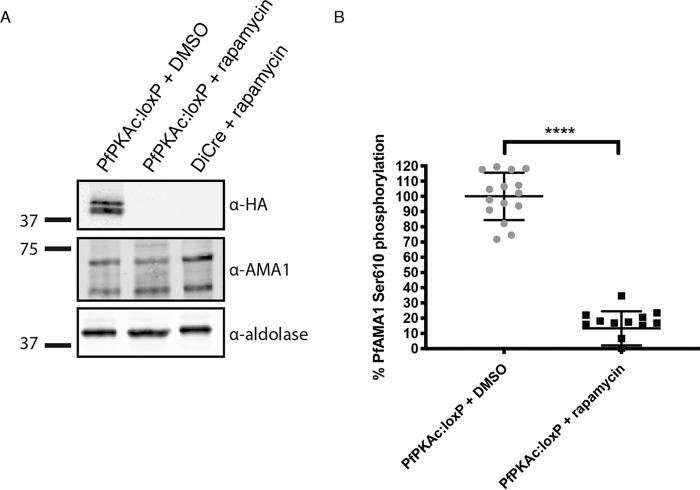
PfPKAc is responsible for PfAMA1 Ser610 phosphorylation. (A) Western blot showing unchanged expression levels of AMA1 following PfPKAc knockdown in PfPKAc:loxP late-schizont-stage parasites. (B) Late-schizont-stage parasites were harvested and lysed following DMSO or rapamycin treatment and incubated with recombinant PfAMA1 cytoplasmic tails. Phosphorylation of Ser610 was detected in an ELISA format using anti-PfAMA1Ser610p antibody (23). Dose-response curves representing cAMP responses were generated, and data were normalized to DMSO-treated controls. Data are represented as average percentages of phosphorylation relative to DMSO-treated controls at 4 μM cAMP. Data are presented as means ± SD; ****, *P* < 0.00005 (unpaired *t* test).

The invasion defect seen following PfPKAc knockdown implied an inability to form a functional moving junction. PfPKAc has previously been implicated in phosphorylation of Ser610 of the short cytoplasmic tail of PfAMA1; however, those studies were performed using recombinant proteins or mammalian PKA homologues (21, 23). Although it has been established that the widely used PKAc inhibitor H89 inhibits native PfAMA1 phosphorylation on Ser610, a direct link to PfPKAc activity has not been shown (35). To confirm that PfPKAc directly phosphorylates PfAMA1 Ser610, we investigated whether PfPKAc-deficient parasite lysate could still phosphorylate the cytoplasmic domain of PfAMA1, using an antibody for phosphorylated PfAMA1 Ser610 (23). Initially, it was shown that PfAMA1 protein levels remained unchanged following PfPKAc knockdown (Fig. 5A). The level of PfPKAc protein knockdown in the parasite lysates used in the phosphorylation assays was consistent across replicates at between 90% and 97% (Fig. S3A). Lysates of DMSO-treated PfPKAc:loxP parasites were able to phosphorylate recombinant PfAMA1 Ser610 in a cAMP-dependent manner; however, a complete loss in cAMP-dependent phosphorylation of AMA1 S610 was observed following PfPKAc knockdown (Fig. S3B). At 4 μM cAMP, expressed as a percentage of DMSO control, there was a significant reduction in levels of PfAMA1 S610 phosphorylation by PfPKAc:loxP parasite lysate compared to the parental strain results, demonstrating that DiCre-mediated excision of PfPKAc leads to a loss of Ser610 phosphorylation in the cytoplasmic domain of AMA1 (Fig. 5B).

10.1128/mBio.01972-19.3 FIG S3 Detection of phosphorylated PfAMA1 at Ser610. (A) Western blot showing similar levels of rapamycin-induced PfPKAc knockdown in each biological replicate used for ELISAs as shown by anti-HA signal. Densitometry plots show >90% protein knockdown compared to DMSO controls in each replicate. (B) Dose-response curves for cAMP-dependent phosphorylation of recombinant PfAMA1 Ser610 by lysates of PfPKAc:loxP parasites treated with DMSO or rapamycin. Download FIG S3, TIF file, 1.9 MB. Copyright © 2019 Wilde et al. 2019 Wilde et al. This content is distributed under the terms of the Creative Commons Attribution 4.0 International license.

## DISCUSSION

Invasion of erythrocytes by P. falciparum merozoites is an extremely rapid, highly dynamic process, and the signaling pathways that trigger the many cellular interactions involved remain mostly unknown. Phosphoproteomic analysis has revealed essential roles for cAMP-dependent phosphorylation as a key posttranslational modification in coordinating the steps of invasion (26, 36, 37). The present study follows another recent publication which also sought to provide the first genetic evidence of the importance of PfPKAc in parasite invasion (26), and our data are fully consistent with those reported from that study.

P. falciparum invasion has been well characterized as being divided into visually distinct stages driven by carefully timed stepwise underlying molecular events. These include initial contact, reorientation, membrane deformation, and internalization of merozoites in under 2 min, followed by echinocytosis of the host erythrocyte and then by the eventual recovery of the cell to its normal shape (33, 34, 38). Live-imaging experiments revealed that PfPKAc-deficient parasites attached to erythrocytes at a lower rate than wild-type (WT) parasites but were still able to make contact with host cells, initiate membrane deformation, and trigger echinocytosis. Echinocytosis has been considered a standard measure for successful invasion; however, the events surrounding invasion, their timing, and what triggers them are being recharacterized with improvements in genetic modification and live-cell microscopy techniques (33, 34). Recent studies have found that echinocytosis is not a hallmark phenotype of invaded erythrocytes, with up to 30% of invasion events not displaying this marker (34). It is therefore unlikely that invasion is essential for echinocytosis or that echinocytosis marks successful invasion. Upon PfPKAc depletion, merozoites could no longer successfully invade and instead remained attached to the erythrocyte surface. This behavior was remarkably similar to what has been seen in several previous studies utilizing video microscopy of parasites in which the AMA1-RON interaction has been blocked, either by gene deletion or by treatment with PfAMA1 inhibitor R1 (22, 34, 39, 40). In a previous study involving DiCre-mediated depletion of PfAMA1, Yap et al. postulated that, following failed attempts by PfAMA1-depleted merozoites to penetrate and invade, the erythrocyte surface cannot be resealed, resulting in prolonged echinocytosis of erythrocytes that cannot recover (40). Those authors speculated that this was due to insufficient PfAMA1 on the surface to form a tightly sealed moving junction around the invading merozoite. However, this prolonged echinocytosis phenotype was also observed in PfPKAc-deficient parasites, suggesting that the mechanisms governing echinocytosis function downstream of invasion. This phenocopy of the PfAMA1 mutant suggests a role for PfPKAc as a “molecular switch” working upstream of the moving junction. It is peculiar that the PfPKAc mutant has such a defined phenotype specific to invasion similar to that seen for PfAMA1 mutants, when phosphoproteomic studies have predicted a prominent role for cAMP-dependent signaling in schizont-stage parasites (36, 37), indicating PfPKA likely has many phosphorylation targets.

A recent study also sought to provide genetic evidence to support the idea of a role for PfPKAc in parasite invasion, utilizing a similar strategy of DiCre-mediated gene regulation (26). The phenotype described by Patel et al. is fully consistent with our data, showing an invasion defect following PfPKAc knockdown. We were able to show a loss of Ser610 phosphorylation on PfAMA1 biochemically, while Patel et al. identified this phosphorylation site in phosphoproteomic experiments. Patel et al. also observed a reduction in shedding of surface PfAMA1 and described a structural change in the cytoplasmic tail of PfAMA1 following Ser610 phosphorylation, suggesting that this structural change might facilitate interaction with enzymes involved in PfAMA1 shedding and that this process might be necessary for successful invasion by fine-tuning the amount of PfAMA1 on the surface (26). Furthermore, the reduced attachment phenotype seen in live-imaging experiments could reflect the presence of fewer adhesins on the merozoite surface, suggesting a possible defect in microneme secretion.

In recent years, mounting evidence has supported the idea of interplay between the cyclic nucleotide and Ca^2+^ signaling pathways in parasite invasion (11, 24, 25). Previous work in T. gondii showed that depletion of TgPKAc1 leads to a dysregulation of cytosolic Ca^2+^, suggesting that cAMP signaling and, in turn, TgPKAc1 play roles in the negative regulation of Ca^2+^ signaling (24). However, Patel et al. found no change in intracellular Ca^2+^ levels following PfPKAc knockdown, suggesting a fundamental difference between the processes of regulation of Ca^2+^ in these two parasites (26). It will be interesting to see how this unfolds, and it is apparent there is complex interplay between different signaling pathways in P. falciparum that needs to be further explored, and efforts should now be focused on dissecting the order of events in relation to cAMP and Ca^2+^ signaling and on how these two pathways interact in parasite invasion. We have identified PfPKAc phosphorylation to be critical for merozoite invasion, and yet the precise function of this molecular event remains unclear. As previously postulated, the current study raises the issue of whether PfAMA1 phosphorylation by PfPKAc functions to trigger the downstream events of invasion or whether it represents an “off switch” that functions to keep merozoites in a repressed state from which to be activated for invasion by a subsequent dephosphorylation step (23, 26). There is a need for better temporal resolution of the molecular events in the invasion process, and we are beginning to see dissection of these events with improvements in phosphoproteomic methods (26, 37). Previous studies in T. gondii have utilized Ca^2+^ biosensors such as GCaMP6 to understand the interplay with Ca^2+^ in the invasion process (11, 24, 41). cAMP biosensors have also emerged, and these tools, applied to P. falciparum, will be of paramount importance in efforts to understand these signaling pathways in malaria infection.

This report highlights not only the essentiality of PfPKAc in P. falciparum but also the importance of protein phosphorylation in invasion. In addition, this report confirms the identity of the kinase responsible for the most extensively studied posttranslational modification in parasite invasion, PfAMA1 Ser610 phosphorylation. In order to design better novel therapies against malaria and other parasitic diseases, it is vital to understand the signaling cascades that govern molecular interactions of invasion, and it is becoming clear that protein phosphorylation may be a good avenue for novel interventions.

## MATERIALS AND METHODS

### Cloning of DNA constructs.

The pCas9-PfPKAc guide plasmid was generated from a previously modified pUF-Cas9 vector (pUF-cas9G) that encodes both Cas9 and single guide RNA (sgRNA) (7, 42). The *BtgZI* adaptor sequence of the modified pUF1-Cas9G vector was replaced with the specific PfPKA guide DNA sequence 5′-CCAGAAATTTTATTGAACGT-3′ using a previously published protocol (42).

pΔPfPKAc was generated by modifying p1.2MSP9-HA-glms (referred to here as p1.2) using a previously described protocol (7). A homologous target sequence corresponding to the 3′ end of *pfpka* was cloned into p1.2 with EcoRI and KasI, producing an intermediate vector. A homologous target sequence corresponding to the 5′ end of *pfpka* was then cloned into BglII/SpeI sites. In this construct, homologous flanks corresponded to upstream and downstream regions of the protospacer-adjacent motif (PAM), thus facilitating homology-directed repair (HR), resulting in the disruption of the *pfpka* locus and rendering the gene inactive.

To generate pPfPKAc:loxP, a synthetic fragment was synthesized (Integrated DNA Technologies, USA) containing *pfpkac* sequence in which the 5′ region upstream of the PAM site corresponded to WT sequence whereas the downstream sequence was codon optimized. A triple-hemagglutinin (HA) epitope was added to the end of codon-optimized *pfpkac.* From this construct, PfPKAc was expressed as a HA fusion protein. A *loxP* site was introduced into an artificial intron prior to the codon-optimized coding sequence, while a second *loxP* site was introduced after the stop codon of HA. The synthetic fragment was cloned into BglII/SpeI sites of the intermediate p1.2 construct used in pΔPfPKAc generation. Downstream of *pfpka* is the human dihydrofolate reductase (*hdhfr*) gene cassette, conferring resistance to antifolate inhibitor WR99210.

To generate parental lines stably expressing DiCre, 2 plasmids were used to introduce the DiCre sequence into the Rh3 pseudogene by CRISPR. The first (Rh3g1) consisted of a 20-bp sequence (5′-AATGATGAAACCTTAGTTGA-3′) targeting the Rh3 gene and cloned into the pUF-cas9G plasmid. The second plasmid (NC5.2) contained Rh3 sequences flanking the cas9 cut site (Rh3 5′ and 3′ flanks for HR). Between these flanks were 3 cassettes carrying the blasticidin (BSD) resistance gene and the Cre19-59 and Cre60-343 regions, respectively.

### Parasite cultures and transfections.

Asexual P. falciparum parasites were cultured in human O^+^ erythrocytes at 4% hematocrit in RPMI medium supplemented with HEPES buffer (25 mM), 0.2% NaHCO_3_, 5% heat-inactivated human serum, and 5% AlbuMAX. Parasites were grown in an atmosphere that included 7% CO_2_, 5% O_2_, and 88% N_2_. Parasites were synchronized using sorbitol as previously described (43). Late-stage schizonts (>40 h postinvasion) were harvested for immunoblotting, Southern blotting, fluorescence-activated cell sorter (FACS) analysis, and microscopy. For light microscopy, parasite culture smears were fixed in 100% methanol and subjected to staining using Giemsa stain (Merck).

NF54 and 3D7 strains were used for transfections. Prior to transfection, highly synchronous mature schizonts were isolated from uninfected erythrocytes by magnetic separation, as previously described (31). Briefly, mature-stage parasites were purified using a magnet-activated cell sorting (MACS) magnetic separation column (Miltenyi Biotec, Gladbach, Germany) and were then incubated in E64 protease inhibitor for 1 h until parasitophorous vacuole (PV)-enclosed merozoite structures (PEMS) could be visualized.

Transfections utilized Amaxa Basic Parasite Nucleofector kit 2 (Lonza, Hilden, Germany). A 100-μg volume of each plasmid was resuspended in 85 μl solution 2 and 15 μl solution 3. This solution was mixed with parasite PEMS and transferred to a 2-mm-gap-size cuvette (Lonza, Hilden, Germany). Transfections were performed using Amaxa 1D Nucleofector and condition U33. Transfected cells were added to 10 ml RPMI-HEPES media at 4% hematocrit and cultured as usual. Blasticidin (BSD; 2.5 μg/ml) or WR99210 (2.5 nM) was added after 24 h to select for positive transfectants.

### Southern blotting.

For *pfpkac* excision analysis, genomic DNA was extracted from parasites using Tris-buffered phenol-chloroform-isoamyl alcohol (25:24:1) and digested with NcoI/AvaII. Digested DNA was fragmented by 1% agarose gel electrophoresis at 14 V overnight and transferred to a Hybond N+ nylon membrane (GE Healthcare). Hybridization was performed using a DIG Easy Hyb kit (Roche) according to the manufacturer’s protocol. Dioxygenin (DIG)-labeled probes were synthesized using a PCR DIG probe synthesis kit (Roche). A probe to label a fragment across the codon-optimized sequence of PfPKAc was synthesized using pPfPKAc:loxP as a template. Following hybridization, membranes were equilibrated, blocked, and incubated with anti-digoxigenin-AP Fab fragments, washed, and equilibrated in detection buffer. Chemiluminescence reagent CSPD was applied to membranes, which were then exposed to X-ray film to enable visualization of DNA.

### Immunoblotting.

Highly synchronous late-stage schizonts were harvested with 0.15% saponin and solubilized in reducing Laemmli sample buffer. Proteins were separated on 4% to 12% Bis-Tris-reducing polyacrylamide gels (Thermo Scientific). Electrophoresed proteins were transferred to nitrocellulose membrane (GE Healthcare Life Sciences). Membranes were blocked in 10% (wt/vol) skim milk–phosphate-buffered saline (PBS)–0.1% Tween 20 and probed with the following primary antibodies: mouse anti-HA (1:500); rabbit anti-HSP70 (1:20,000); rabbit anti-AMA1 R1072 (1:500); and rabbit anti-aldolase (1:500). Horseradish peroxidase (HRP)-conjugated secondary antibodies were used at 1:1,000. Immunoblots were visualized with enhanced chemiluminescence (Thermo Scientific) and X-ray film.

### Parasite growth assays.

Highly synchronous ring-stage NF54 WT, DiCre, or PfPKAc:loxP parasites were plated in triplicate at 1% parasitemia and 4% hematocrit. Parasites were incubated for 72 h in the presence of DMSO or rapamycin (300 nM in 0.1% [vol/vol] DMSO).

For multicycle growth assays, parasites were plated at 1%, 0.1%, 0.01%, or 0.001% parasitemia and incubated with rapamycin or DMSO for 72, 96, 120, or 144 h, respectively. Parasites were stained with ethidium bromide (1:100)–PBS for 10 min. Cells were washed and resuspended in PBS, and growth was determined by fluorescence-activated cell sorter (FACS) analysis as the percentage of parasitemia with respect to the level determined for control parasites.

### Immunofluorescence assay (IFA).

Highly synchronous mature schizonts were isolated from uninfected erythrocytes using a MACS magnetic separation column (Miltenyi Biotec, Gladbach, Germay). Parasites were fixed and stained for IFA as previously described (44). Briefly, parasites were washed once in PBS and then fixed in 4% paraformaldehyde (Sigma-Aldrich, MO)–0.0075% glutaraldehyde (EMS, PA)–PBS for 30 min. Cells were washed in PBS and permeabilized in 0.1% Triton X-100–PBS for 10 min. Cells were washed again and blocked for 1 h with 3% bovine serum albumin (BSA)–PBS and then incubated with rabbit anti-GAP45 R728 (1:200) and rat anti-HA 3F10 (1:1,000) for 1 h. Cells were washed and then probed for 1 h with Alexa Fluor-conjugated secondary antibodies (Invitrogen) for 1 h. Cells were settled onto coverslips coated with 1% polyethylenimine (Sigma-Aldrich, MO). Cells were washed and mounted onto glass microscope slides with Vectashield (Vector Labs) plus 5 μg/ml DAPI (4′,6-diamidino-2-phenylindole). Parasites were imaged on a Zeiss LSM 880 laser scanning microscope.

### Live-cell imaging.

Cultures (30 ml) of highly synchronous 3D7 WT or NF54 PKAloxP ring-stage parasites were prepared and treated with either DMSO (Sigma-Aldrich, MO) or rapamycin (LC Laboratories, MA). Once parasites had developed to become late schizonts, parasites were isolated from uninfected erythrocytes using a MACS magnetic separation column (Miltenyi Biotec, Gladbach, Germay). Cultures were centrifuged at 500 relative centrifugal force (RCF) for 5 min, and the parasite pellet was diluted 1:1,000 in fresh RPMI-HEPES. A 200-μl volume of RPMI-HEPES (0.25% hematocrit) was placed into microscope viewing chambers (Ibidi). A 50-μl volume of parasite suspension was applied to the fresh erythrocytes just prior to imaging. Parasites were imaged on an inverted Zeiss Live Cell AxioObserver microscope under bright-field conditions. The sample chamber was heated to 37°C and supplied with a humidified atmosphere that included 1% O_2_, 5% CO_2_, and 94% N_2_. Quantitation of live-imaging experiments was performed using Image J software.

### Invasion assay.

Merozoites were purified as previously described (31). Briefly, highly synchronized ring-stage PfPKAc:loxP parasites were treated with DMSO or rapamycin and grown until they became late-stage schizonts. Mature schizonts were isolated from uninfected erythrocytes using a MACS magnetic separation column (Miltenyi Biotec, Gladbach, Germay). Schizonts were treated with E64 cysteine protease inhibitor (10 μM) for 4–6 h to prevent egress. Merozoites were purified from schizont preparations by passage through a 1.2-μm-pore-size syringe filter (Acrodisc) (32 mm, Pall). Filtrate containing purified merozoites was added to uninfected erythrocytes (final concentration, 1% hematocrit), and suspensions were agitated at 37°C for 20 min. Suspensions were then plated out in triplicate (100 μl per well, topped up with media to total 200 μl). After 24 to 40 h, levels of parasitemia were determined by flow cytometry as described above. The concentrations of merozoites and erythrocytes were quantitated using CountBright Absolute counting beads (Life Technologies), and the invasion rate was calculated as follows: percentage of erythrocytes invaded × [(number of erythrocytes per microliter)/(number of merozoites per microliter)] (31).

### Phosphorylation assay.

Late-schizont-stage parasites grown in the presence of DMSO or rapamycin were isolated as described above and lysed in a mixture containing 20 mM Tris (pH 7.4), 1% Triton, 150 mM NaCl, 20 mM MgCl_2_, 1 mM ATP, and 1 mM dithiothreitol (DTT) complemented with Complete protease inhibitor and PhosSTOP phosphatase inhibitor (Roche). Nunc MaxiSorp 96-well enzyme-linked immunosorbent assay (ELISA) plates were coated with a WT glutathione *S*-transferase (GST)–PfAMA1 tail fusion protein at 1 μg/ml and blocked with 1% BSA–Tris-buffered saline (TBS) (23). Cleared parasite lysates were added to wells along with serial dilutions of cAMP, and plates were incubated at 37°C for 30 min. After washing with TBS, wells were probed with 1 μg/ml rabbit anti-PfAMA1Ser610_p_ for 1 h (23). Rabbit anti-PfAMA1Ser610_p_ was then detected with 0.2 μg/ml HRP-conjugated goat anti-rabbit antibody. Assays were developed with tetramethylbenzidine substrate (Thermo Scientific) followed by an equal volume of 2 M HCl, and plates were read at 405 nm on a spectrophotometer.
