# Supplementary figures and images for: Protein Kinase A Is Essential for Invasion of Plasmodium falciparum into Human Erythrocytes
Source: mBio. 2019 Oct 8;10(5):e01972-19. doi: 10.1128/mBio.01972-19 (PMC6786871; doi:10.1128/mBio.01972-19)

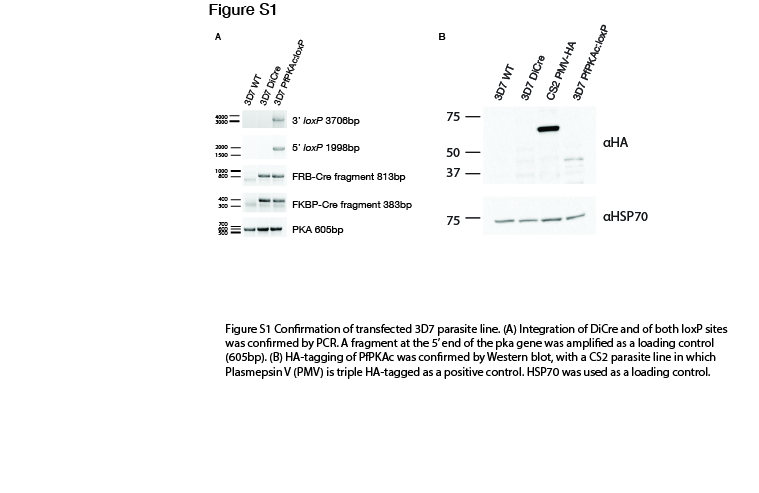

Supplement: FIG S1 [file mBio.01972-19-sf001.tif]

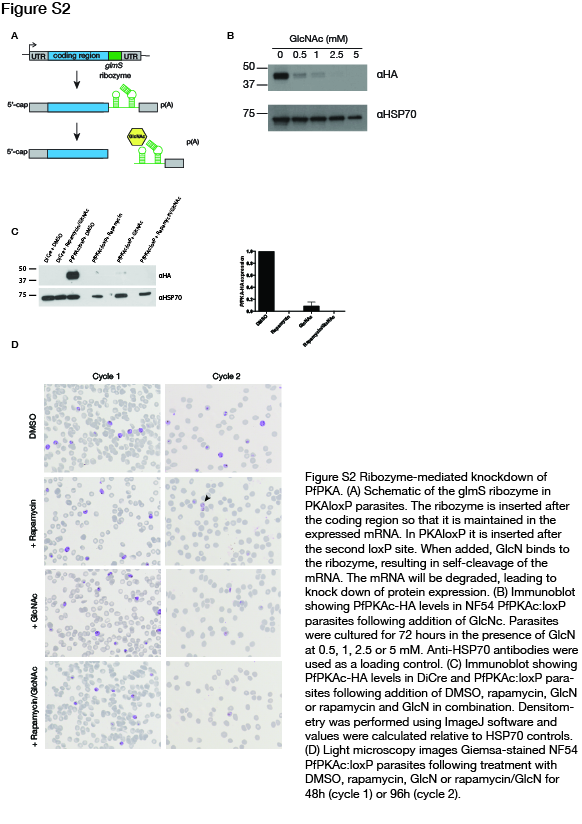

Supplement: FIG S2 [file mBio.01972-19-sf002.tif]

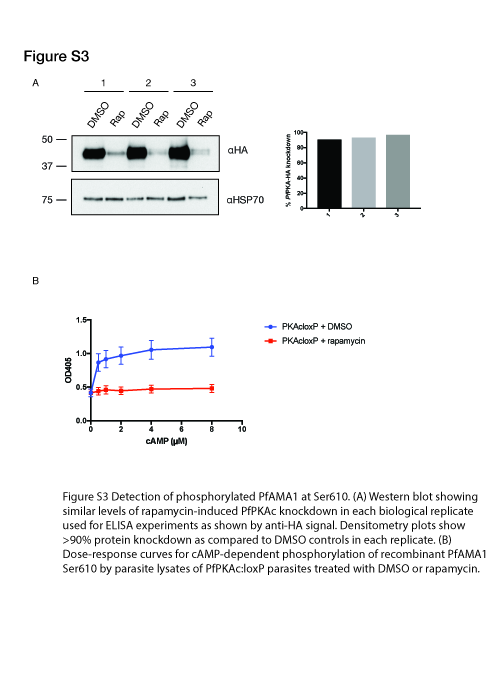

Supplement: FIG S3 [file mBio.01972-19-sf003.tif]
